# A novel, cassette-based nitric oxide delivery system with an advanced feedback control algorithm accurately delivers nitric oxide via the anesthesia machine independent of fresh gas flow rate and volatile anesthetic agent

**DOI:** 10.1007/s10877-024-01143-4

**Published:** 2024-06-01

**Authors:** Mark D. Twite, Aaron W. Roebuck, Stephanie R. Anderson

**Affiliations:** 1 Department of Anesthesiology, Children’s Hospital Colorado & University of Colorado Anschutz Medical Campus, 13123 East 16th Avenue, Box 090, Aurora, CO 80045 USA; 2 Department of Clinical Services, Vero Biotech Inc, Atlanta, GA USA; 3 Department of Applications Engineering, Vero Biotech Inc, Atlanta, GA USA

**Keywords:** Nitric oxide, Anesthesia machine, Low-flow anesthesia

## Abstract

Nitric oxide (NO), a selective pulmonary vasodilator, can be delivered via conventional ICU and anesthesia machine ventilators. Anesthesia machines are designed for rebreathing of circulating gases, reducing volatile anesthetic agent quantity used. Current cylinder- and ionizing-based NO delivery technologies use breathing circuit flow to determine NO delivery and do not account for recirculated gases; therefore, they cannot accurately dose NO at FGF below patient minute ventilation (MV). A novel, cassette-based NO delivery system (GENOSYL^®^ DS, Vero Biotech Inc.) uses measured NO concentration in the breathing circuit as an input to an advanced feedback control algorithm, providing accurate NO delivery regardless of FGF and recirculation of gases. This study evaluated GENOSYL^®^ DS accuracy with different anesthesia machines, ventilation parameters, FGFs, and volatile anesthetics. GENOSYL^®^ DS was tested with GE Aisys and Dräger Fabius anesthesia machines to determine NO dose accuracy with FGF < patient MV, and with a Getinge Flow-i anesthesia machine to determine NO dose accuracy when delivering various volatile anesthetic agents. Neonatal and adult mechanical ventilation parameters and circuits were used. GENOSYL® DS maintained accurate NO delivery with all three anesthesia machines, at low FGF with recirculation of gases, and with all volatile anesthetic agents at different concentrations. Measured NO_2_ levels remained acceptable at ≤ 1 ppm with set NO dose ≤ 40 ppm. GENOSYL^®^ DS, with its advanced feedback control algorithm, is the only NO delivery system capable of accurately dosing NO with anesthesia machines with rebreathing ventilation parameters (FGF < MV) regardless of anesthetic agent.

## Introduction

Nitric oxide (NO) gas is a molecule that plays a pivotal role in many physiological processes. In the cardiovascular system, NO is released by the endothelial cell and acts as a vascular smooth-muscle relaxant to induce systemic and pulmonary vasodilation [1]. NO is rapidly inactivated by oxyhemoglobin; therefore, inhaled NO (iNO) acts as a pure pulmonary vasodilator with negligible systemic hemodynamic effects [2]. Studies have demonstrated that iNO in neonatal patients with hypoxic respiratory failure who had clinical or echocardiographic evidence of pulmonary hypertension improves oxygenation and reduces the need for extracorporeal membrane oxygenation. [3, 4]

Many patients who are receiving NO through a conventional intensive care unit (ICU) ventilator will require general anesthesia in an operating room or cardiac catheterization laboratory; alternatively, many patients undergoing general anesthesia will require initiation of NO therapy—for example, during complex cardiac surgery in newborns. Therefore, NO delivery should integrate seamlessly into both the conventional ICU ventilator and the anesthesia machine ventilator, which is challenging because of fundamental differences between these systems (Table 1). Rebreathing does not occur with the conventional ICU ventilator open-circuit design, in which air and oxygen flow continuously through the circuit throughout the breathing cycle and exhaled gases are vented to the atmosphere. In contrast, the anesthesia circuit is a semi-closed circuit, allowing for rebreathing when fresh gas flow (FGF) is less than minute ventilation (MV) [5, 6]. Anesthesia circuits contain a carbon dioxide (CO_2_) absorber and gas reservoir system that are essential for rebreathing to occur safely. The anesthesia semi-closed circuit was primarily designed to conserve volatile anesthetic agent use by allowing low FGFs [7, 8].

**Table 1:** Properties of the anesthesia machine ventilator and ICU ventilator

Anesthesia machine ventilator	ICU ventilator
*Circle system (variable degrees of rebreathing)*	*Open system*
Designed for low FGF	High FGF of air/oxygen only
Conservation of inhalational agent	Exhaust gases to the room via filter
Scavenging of waste gas required	No need for scavenging system
Carbon dioxide absorber required	Delivers only mechanical ventilation
Manual or mechanical ventilation	Operator directly sets the FiO_2_
FiO_2_ determined by flow meters or set control	
Delivers O_2_ and other anesthetic gases (and NO)	Delivers only O_2_ and air (and NO)
HME filters	Heated, humidified circuits

Adapted with permission from Austin PN, et al. (6) Respir Care. 66(7):1184–1195

*FGF* fresh gas flow, *FiO*_*2*_ fraction of inspired oxygen, *HME* heat and moisture exchangers, *NO* nitric oxide, *O*_*2*_ oxygen

In the modern era, there has been a move toward low-flow anesthesia to take further advantage of this semi-closed circuit on anesthesia machines. A practical definition of low-flow anesthesia is the reduction of FGF below patient MV to the lowest level consistent with equipment capabilities and provider comfort while ensuring safe and effective care for the patient [9]. The benefits of low-flow anesthesia include conserving humidity and temperature in the lungs to maintain a more physiologic environment in the respiratory tract during mechanical ventilation [10–12] and better preservation of respiratory function and mucociliary clearance [13]. Maintaining the temperature, humidity, and respiratory function is particularly important at the extremes of patient age and during long cases requiring general anesthesia [14]. Low-flow anesthesia also reduces volatile anesthetic agent use, which decreases cost as well as greenhouse gas effects from waste gases scavenged from the anesthesia machine [15, 16].

Currently, there are three commercially available technologies for NO delivery systems: cylinder-based systems, an ionization system, and a cassette-based system. The cylinder- and ionization-based technologies use breathing circuit flow measurements to deliver NO. Although both systems measure and display the NO delivered to the patient, this measured value is not used to adjust the delivered NO dose; therefore, these systems cannot deliver NO accurately under rebreathing conditions with the anesthesia machine ventilator.

Cylinder-based NO delivery technology relies on pressurized cylinders containing high-dose concentrations of NO buffered with an inert gas such as nitrogen to avoid the generation of toxic nitrogen dioxide (NO_2_) within the cylinder. This pressurized cylinder is connected to a flow-regulated injector module that delivers NO into the inspiratory limb of a ventilator circuit based on breathing circuit flow. Ionization-based NO delivery technology generates NO from air and delivers NO into the inspiratory limb of a ventilator circuit based on breathing circuit flow. Several studies using flow-based NO delivery systems with anesthesia machines have concluded that NO delivery to the patient is accurate only when FGF is equal to or greater than MV [17–19]. Because flow-based NO delivery systems do not account for recirculating gases in semi-closed circuits on an anesthesia machine, the many benefits of low-flow anesthesia are negated. Further, flow-based NO delivery systems introduce a potential error in NO delivery if the anesthesia provider is not aware that FGF must be equal to or greater than MV to prevent recirculation of gases when using these systems. In addition, volatile anesthetic agents may also affect the accuracy of measured NO concentration in flow-based NO delivery systems [20].

**Fig. 1:**
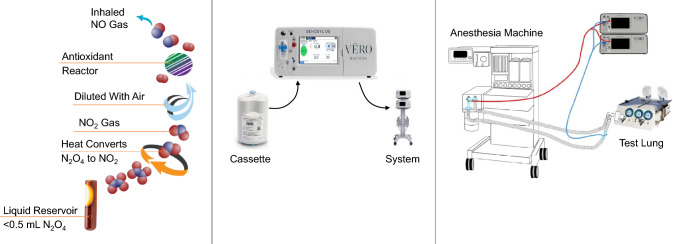
NO generation pathway, the GENOSYL^®^ DS, and experimental setup. **a** The N_2_O_4_ to NO chemical conversion. **b** GENOSYL^®^ DS measures NO concentration in the breathing circuit and uses the measured value in an advanced feedback-control algorithm to accurately determine how much NO should be injected when rebreathing in semi-closed anesthesia circuits. **c** Anesthesia experimental setup used a dual-limb anesthesia circuit with injection port at the inspiratory output of the machine. The sampling port was 6 to 12 inches from the patient wye on the inspiratory limb of the breathing circuit. *DS* delivery system, *N*_2_*O*_4_ dinitrogen tetroxide, *NO* nitric oxide

The GENOSYL^®^ Delivery System (DS) cassette-based technology was approved by the FDA in 2019. The GENOSYL^®^ DS is the only NO delivery system that uses the measured NO concentration in the breathing circuit as the input to an advanced feedback control algorithm, to accurately dose NO. Its advanced feedback control algorithm allows accurate NO delivery to the patient with both an open breathing circuit on a conventional ICU ventilator and a semi-closed circuit on an anesthesia machine ventilator, even under rebreathing conditions. Thus, the GENOSYL^®^ DS does not require FGF to be greater than or equal to MV. The GENOSYL^®^ DS generates on-demand NO through a two-step process [21, 22]. The first step is the generation of NO_2_ from the vaporization of liquid dinitrogen tetroxide (N_2_O_4_), and the second step is the conversion of NO_2_ to NO using ascorbic acid (Fig. 1). This study tested the accuracy of the GENOSYL^®^ DS under multiple conditions that reflect clinical anesthesia practice, including rebreathing conditions.

## Methods

### Study design

The aim of the study was to test the accuracy of GENOSYL^®^ DS delivery of NO with its advanced feedback control algorithm when using semi-closed circuits with different anesthesia machines. The specific study objectives were to evaluate:

1. NO delivery accuracy with FGF less than and greater than MV using neonatal and adult anesthesia circuits and different ventilation settings;
2. NO delivery accuracy with three volatile anesthetic agents (sevoflurane, isoflurane, and desflurane) and nitrous oxide (N_2_O); and
3. Whether NO_2_ remains within acceptable limits.

### Procedures

The study objectives were tested using two different protocols.

#### Protocol 1

To assess objectives 1 and 3, GENOSYL^®^ DS was tested with GE Aisys CS2 and Dräger Fabius GS Premium anesthesia machines using dual-limb neonatal and adult anesthesia circuits with a Michigan Instruments test lung. NO was injected into the inspiratory limb of the circuit at the anesthesia machine’s inspiratory outlet and sampled on the inspiratory limb 6 to 12 inches from the patient wye. Set NO doses of 1, 20, 40, and 80 ppm were tested in volume control and pressure control mechanical ventilation modes with ventilator settings achieving a MV range of 0.9 to 7.0 L/min for rebreathing test cases and 0.7 to 7.4 L/min for non-rebreathing test cases (Table 2). FGF was adjusted from 0.5 to 2 L/min to assess rebreathing ventilation conditions, and from MV up to approximately 15.0 L/min to assess non-rebreathing ventilation conditions. Manual ventilation was also tested for all set NO doses. Set NO dose and measured NO concentration in the circuit were recorded. NO delivery accuracy was calculated as measured NO concentration minus set NO dose. In this study, the acceptable NO limits were ± 20% or ± 2 ppm (whichever is greater) of the set NO dose, per FDA guidance for NO delivery devices [23]. Standard heat and moisture exchange (HME) filters and antibacterial filters were incorporated into the breathing circuit (Fig. 1).

**Table 2:** Ventilator settings for the different test conditions

Test case	Raw	Cl	Mode	RR	Vt	I:E	PEEP	Pi
	(cm H_2_O/L/s)	(L/cm H_2_O)		(bpm)	(mL)		(cm H_2_O)	(cm H_2_O)
Ventilation settings for non-rebreathing nitric oxide accuracy testing								
Neonatal settings	200	0.003	Pressure control	60		1:2	0	20
				30		1:1	20	30
			Volume control	60	30	1:1	0	
				30	40	1:2	20	
			Manual	30	40	1:2	20	
Pediatric/Adult settings	20	0.015	Pressure control	6		1:4	0	40–50
				6		1:2	20	40–65
			Volume control	6	500	1:2	20	
				6	700	1:4	0	
			Manual	10	700	1:2	0	
Ventilation settings for rebreathing nitric oxide accuracy testing								
Neonatal settings	200	0.003	Pressure control	60		1:2	0	20
				30		1:2	20	30–50
			Volume control	60	30	1:2	0	
				30	45–50	1:2	5	
			Manual	30	45–50	1:2	5	
Pediatric/Adult settings	20	0.015	Pressure control	6		1:4	0	40–50
				6		1:2	20	40–65
			Volume control	6	500	1:2	20	
				6	700	1:4	0	
			Manual	10	700	1:2	0	

*Cl* lung compliance, *I:E* inspired to expired ratio, *PEEP* positive end-expiratory pressure, *Pi* inspiratory pressure, *Raw* airway resistance, *RR* respiratory rate, *Vt* tidal volume

All test conditions used a set fraction of inspired oxygen (FiO_2_) of 0.6. Concentrations of NO_2_ were assessed for acceptable limits based on FDA guidance for NO delivery devices [23].

#### Protocol 2

To assess objectives 2 and 3, GENOSYL^®^ DS was tested with a Getinge Flow-i anesthesia machine using dual-limb neonatal and adult anesthesia circuits with a test lung. A 20 ppm set NO dose was used in conjunction with N_2_O and three different volatile anesthetic agents at different minimum alveolar concentration (MAC) values adjusted in 0.5 MAC increments up to 2 MAC. The volatile anesthetic agents used were isoflurane, sevoflurane, and desflurane, which have MAC values of 1.2, 1.9, and 6.5 vol%, respectively, based on 40-year-old adult values [24]. Neonatal and adult mechanical ventilation settings used pressure control and volume control ventilation modes, respectively. Ventilator settings were adjusted to achieve MVs of 0.9 and 5 L/min for neonatal and adult test cases, respectively. FGF was adjusted from 0.5 to 2 L/min for neonatal test settings and 2 to 8 L/min for adult test settings to assess NO delivery accuracy under rebreathing and non-rebreathing ventilation conditions. Set NO dose and measured NO concentration in the circuit were recorded. The acceptable NO limits were defined as a measured NO concentration ± 20% of set NO dose [23]. Standard HME and antibacterial filters were incorporated into the breathing circuit (Fig. 1). All test conditions used a set FiO_2_ of 1.0, except N_2_O test conditions, which used an FiO_2_ of 0.5. NO_2_ levels were assessed for acceptability based on FDA guidance for NO delivery devices [23].

## Results

### NO delivery is accurate with FGF less than and greater than MV

**Fig. 2:**
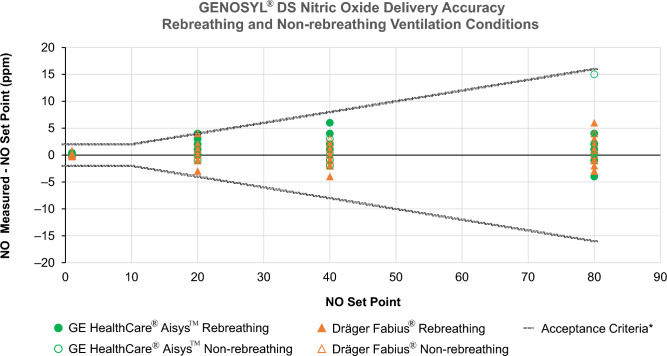
Accuracy of NO delivery using GENOSYL^®^ DS in conjunction with GE and Dräger anesthesia machines in mechanical and manual ventilation modes. *Acceptance criteria were based on guidance for industry [23]. *DS* delivery system, *NO* nitric oxide

In the set NO dose range of 1 to 80 ppm, the GENOSYL^®^ DS maintained accurate NO delivery within ±20% or ±2 ppm (whichever is greater) of set NO dose for both neonatal and adult circuits with non-rebreathing (FGF > MV) and rebreathing (FGF < MV) ventilation conditions in mechanical pressure control and volume control and manual ventilation modes (Fig. 2). Measured NO_2_ levels remained acceptable at ≤1 ppm under all ventilation conditions when set NO dose was ≤40 ppm and set FiO_2_ was 0.6.

### NO delivery is accurate when delivered with volatile anesthetic agents and N_2_O

**Fig. 3:**
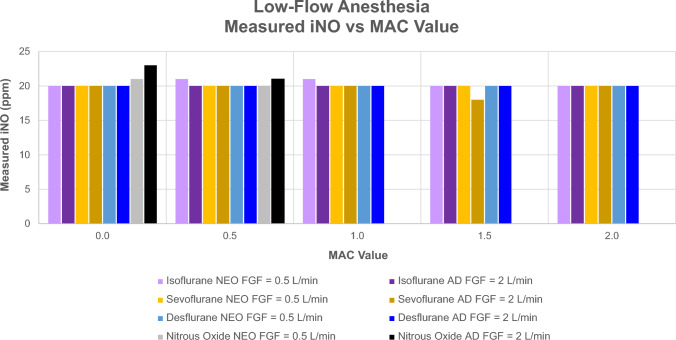
Accuracy of measured NO in neonatal and adult circuits using a Flow-i anesthesia machine in the absence of anesthetic agents and with different anesthetic agents and concentrations. The NO measurement displayed on the Genosyl^®^ DS was recorded 10 min after anesthetic concentration changes. *AD* adult, *FGF* fresh gas flow, *iNO* inhaled NO, *MAC* minimum alveolar concentration, *NEO*, neonatal, *NO* nitric oxide, *N*_*2*_*O* nitrous oxide

The GENOSYL^®^ DS maintained NO delivery within ±20% of the 20 ppm set NO dose when isoflurane, sevoflurane, and desflurane concentrations were incrementally increased and stepped up to 2 MAC and with a 50% N_2_O/50% O_2_ composition. This accuracy was maintained regardless of FGF and MV in both neonatal and adult circuits with non-rebreathing and rebreathing ventilation conditions (Fig. 3). With an FiO_2_ of 1.0, measured NO_2_ levels remained ≤0.2 ppm, which is below the allowed threshold of 1 ppm [23].

## Discussion

These results show that the cassette-based GENOSYL^®^ DS, with its advanced feedback control algorithm, accurately delivered set NO dose via an anesthesia machine ventilator independent of breathing circuit size, mode of ventilation, MV, FGF, ventilator settings, and anesthetic agent type and dose. In addition, NO_2_ production remained within acceptable limits for patient safety as per FDA guidance for NO delivery devices [23]. These data have three important implications:

### Accurate NO delivery with anesthesia machines under rebreathing ventilation conditions

The GENOSYL^®^ DS allows for the continued use of low-flow anesthesia when NO is introduced to the anesthesia circuit, maintaining the benefits of using low-flow anesthesia for the patient, as well as conservation of volatile anesthetic agents. The highly fluorinated volatile anesthetic agents sevoflurane, desflurane, and isoflurane, as well as N_2_O, are greenhouse gases, ozone-depleting agents, or both. These agents undergo minimal metabolism in the body during use and are eliminated unchanged via exhalation and then scavenged to the atmosphere [25]. The global warming effects of these gases vary, with atmospheric lifetimes of 1 to 5 years for sevoflurane, 3 to 6 years for isoflurane, 9 to 21 years for desflurane, and 114 years for N_2_O [25]. Focused programs encouraging low-flow anesthesia have been shown to reduce CO_2_ equivalent emissions by 64% [16]. Many hospitals have designed quality improvement programs to encourage anesthesiologists to turn down their FGF from the traditional 2 L/min to < 1 L/min [26]. The GENOSYL^®^ DS is the only NO delivery system that accurately delivers NO with anesthesia machines under low FGF rebreathing conditions.

### Improved workflow in the perioperative environment

Patients receiving NO are frequently transitioned between the ICU and operative areas. The ability of the anesthesia provider to set the anesthesia machine ventilator, low FGF, and volatile anesthetic agent at the required levels, regardless of the NO delivery device, decreases cognitive workload and distractions. Further, the ability of the GENOSYL^®^ DS to deliver NO accurately with various ventilation devices, including those ventilators used for transport to and from the ICU and anesthesia machine ventilators, enables hospitals to have a single NO delivery system for all areas.

### “Future-proofing” capital investment in a NO delivery system

Low-flow anesthetic techniques require repeated adjustment of the volatile anesthetic agent concentration added to the FGF. New anesthesia machine technology automates this process, which decreases the workload for the anesthesia provider and improves efficiency [27–31]. As modern anesthesia machines increasingly feature this automated technology that targets very low FGF, it will become important for any NO delivery system to be able to deliver NO accurately into a wide range of FGFs. The GENOSYL^®^ DS is the only NO delivery system compatible with current and future anesthesia machine technologies that automate the process of targeting a set end-tidal anesthetic agent concentration at very low FGF.

There are two limitations to this study: First, a limited number of anesthesia machines were tested based on availability during the COVID pandemic, and for experiments involving volatile anesthetic agent, selection was based on availability of vaporizers for all anesthetic agents tested. The study was conducted with three different makes of anesthesia machines; therefore, the results may not apply to all anesthesia machines. The principles of operation of anesthesia machines do differ [32]; but because the GENOSYL^®^ DS measures NO concentration being delivered to the patient—instead of measuring flow through the anesthesia machine circuit—it should be expected to deliver NO accurately independent of the type of anesthesia machine. Second, the study was conducted in vitro, and gas uptake and release were not part of the simulation; it is thus possible that having a patient attached to the anesthesia machine while delivering NO via the anesthesia ventilator circuit could affect the measured NO results. However, the GENOSYL^®^ DS, with its advanced feedback control algorithm, is expected to deliver NO accurately in all clinical situations. In addition, as the pharmacokinetics of the volatile anesthetic agents are well known and consistent in humans, and the properties of NO are also well understood, it is unlikely that in vivo testing would change the conclusions of this study. Potential next steps for investigation are to repeat certain aspects of the study in a clinical environment.

## Conclusions

This study demonstrates that the cassette-based GENOSYL^®^ DS technology, with its advanced feedback control algorithm, is the only NO delivery system capable of accurately delivering NO with an anesthesia machine under rebreathing ventilation conditions and with all widely used volatile anesthetic agents and N_2_O. This accurate delivery is achieved by injecting NO based on measured concentration in the inspiratory circuit limb, rather than breathing circuit flow. This NO delivery system maintains the many benefits of low-flow anesthesia to the patient and keeps anesthetic agent use and waste low, with both cost and environmental benefits.

## Data Availability

Please contact the corresponding author if you would like access to the datasets used or analyzed in this research.
